# MRAP2 regulates ghrelin receptor signaling and hunger sensing

**DOI:** 10.1038/s41467-017-00747-6

**Published:** 2017-09-28

**Authors:** Dollada Srisai, Terry C. Yin, Abigail A. Lee, Alix A. J. Rouault, Nicole A. Pearson, Justin L. Grobe, Julien A. Sebag

**Affiliations:** 1 Department of Molecular Physiology and Biophysics, University of Iowa, Carver College of Medicine, Iowa City, IA 52242 USA; 2 F.O.E.D.R.C, Iowa City, IA 52242 USA; 3 Pappajohn Biomedical Institute, Iowa City, IA 52242 USA; 4 Department of Pharmacology, University of Iowa, Carver College of Medicine, Iowa City, IA 52242 USA

## Abstract

Ghrelin is the only known circulating orexigenic hormone. It is primarily secreted by the stomach and acts at its receptor, the growth hormone secretagogue receptor 1a (GHSR1a), in the hypothalamus to signal hunger and promote food intake. The melanocortin receptor accessory protein 2 (MRAP2) was previously shown to regulate energy homeostasis through the modulation of the activity of the melanocortin-4 receptor and prokineticin receptors. In this study we identify MRAP2 as a partner of ghrelin-GHSR1a signaling. We show that MRAP2 interacts with GHSR1a and potentiates ghrelin-stimulated signaling both in vitro and in vivo. We demonstrate that in the absence of MRAP2, fasting fails to activate agouti-related protein neurons. In addition, we show that the orexigenic effect of ghrelin is lost in mice lacking *MRAP2*. Our results suggest that MRAP2 is an important modulator of the energy homeostasis machinery that operates through the regulation of multiple GPCRs throughout the hypothalamus.

## Abstract (web summary)

Melanocortin receptor accessory protein 2 (MRAP2) is an adaptor protein that contributes to melanocortin-4 receptor and prokineticin receptor 1 signalling. Here the authors show that MRAP2 also regulates ghrelin receptor signalling in the hypothalamus and starvation sensing in mice.

## Introduction

Food intake and energy expenditure are tightly regulated by numerous hormones and neuropeptides secreted either by peripheral organs or centrally^1, 2^. These molecules act on the hypothalamus, through their respective receptors, to inform the brain on the energy state of the organism. Ghrelin is a peptide hormone secreted by the stomach^3^ during fasting to signal hunger to the brain^4, 5^. Ghrelin acts through its receptor, the growth hormone secretagogue receptor 1a (GHSR1a), a G-protein-coupled receptor (GPCR) expressed in the hypothalamus, in particular in orexigenic agouti-related protein (AGRP) neurons^5–8^. Ghrelin function has been shown to be largely mediated through and to require AGRP neurons^5, 9^. Whereas several GPCRs have been shown to be regulated by or to require the presence of accessory proteins, the interaction of GHSR1a with accessory proteins has not been investigated.

The melanocortin receptor accessory protein 2 (MRAP2) is a single transmembrane protein that plays an important role in the control of energy homeostasis since the deletion of MRAP2 in mice causes severe obesity^10^. Mutations in MRAP2 have also been identified in obese humans^10^. MRAP2 is highly expressed in the hypothalamus where it is known to regulate the activity of the melanocortin-4 receptor^10, 11^ and the prokineticin receptors^12^, GPCRs, that signal satiety and are required for the proper control of food intake and energy expenditure^12–16^. In this study we investigated if MRAP2 modulates ghrelin signaling in vitro and in vivo, and conclude that MRAP2 interacts with GHSR1a and is an important component of the ghrelin receptor signaling complex, thus establishing that in addition to being a regulator of satiety sensing through the modulation of MC4R and PKR1 activity, MRAP2 promotes hunger sensing by potentiating ghrelin signaling.

## Results

### MRAP2 deletion reduces physiological response to starvation

**Fig. 1:**
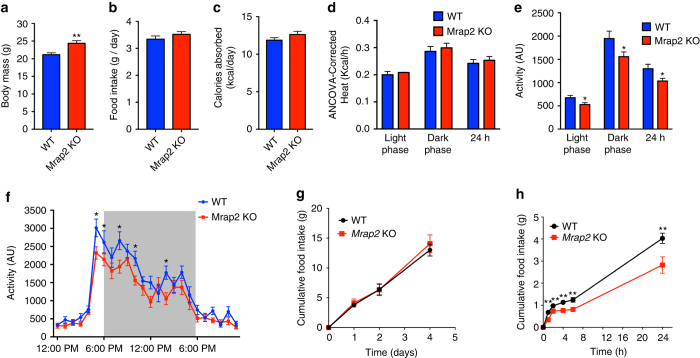
Mrap2 KO mice display decreased locomotor activity. **a** Body mass of 12- to 13-week-old WT (*n* = 12) and *Mrap2* KO (*n* = 13) male mice. **b** Daily food intake of WT and *Mrap2* KO mice. **c** Daily calories absorbed by WT and *Mrap2* KO mice. **d** ANCOVA-corrected energy expenditure of WT and *Mrap2* KO mice. **e** Activity of WT and *Mrap2* KO mice. **f** Hourly activity of WT and *Mrap2* KO mice. **g** Cumulative food intake (regular diet) of WT and *Mrap2* KO mice over 5 days. **h** Cumulative food intake (regular diet) of WT and *Mrap2* KO mice after a 24 h fast. Error bars are SEM. *P < 0.05, **P < 0.01

As previously reported, *Mrap2* knockout (KO) mice develop obesity and their weight start diverging from wild-type (WT) animals between 9 and 11 weeks of age^10^. To identify the cause of the obesity phenotype, we investigated how deleting MRAP2 impacts energy homeostasis in 11- to 13-week-old mice. At this age, *Mrap2* KO animals were slightly but significantly heavier than their WT littermates (Fig. 1a). *Mrap2* KO mice displayed no increase in daily food intake (Fig. 1b), digestive efficiency (Fig. 1c), or heat production (Fig. 1d). However, we found that *Mrap2* KO mice have a significant decrease in locomotor activity compared to WT littermates (Fig. 1e, f), suggesting that the obesity caused by MRAP2 deletion is, at least in part, due to decreased activity. While we observed no significant difference in daily food intake (Fig. 1b) or accumulated food intake (Fig. 1g) between WT and *Mrap2* KO mice, we found that mice lacking MRAP2 eat about 30% less food compared to their WT littermates in the day following a 24 h fast (Fig. 1h), thus suggesting that MRAP2 is important for hunger sensing and that *Mrap2* KO mice experience a weaker drive to eat following a fast.

**Fig. 2:**
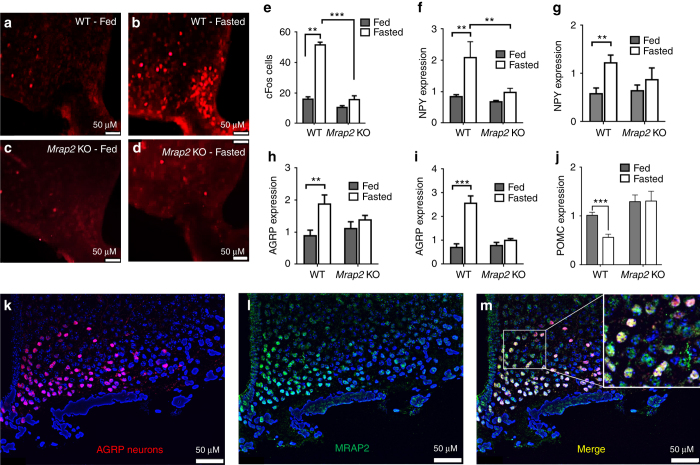
MRAP2 is important for the stimulation of AGRP neurons by fasting. **a**–**d** cFos expression in the arcuate nucleus of fed WT (**a**), 24 h-fasted WT (**b**), fed *Mrap2* KO (**c**), and 24 h-fasted *Mrap2* KO (**d**) mice. **e** Quantitative measurement of cFos activation in the arcuate nucleus of fed or fasted WT and *Mrap2* KO mice. **f**, **g** NPY expression measured by qPCR in the arcuate nucleus of male (**f**) and female (**g**) fed or 24 h-fasted WT and *Mrap2* KO mice. **h**, **i** AGRP expression measured by qPCR in the arcuate nucleus of male (**h**) and female (**i**) fed or 24 h-fasted WT and *Mrap2* KO mice. **j** POMC expression measured by qPCR in the arcuate nucleus of male fed or 24 h-fasted WT and *Mrap2* KO mice. **k**–**m** Colocalization of AGRP (**k**) and MRAP2 (**l**) by immunofluorescence microscopy in the arcuate nucleus. AGRP neurons expressing MRAP2 are shown in *yellow* in the merged image (**m**). Error bars are SEM. ***p < 0.001, **p < 0.01

The hypothalamus plays a critical role in the control of energy homeostasis, and the orexigenic AGRP neurons of the arcuate nucleus (ARC) are essential for sensing energy needs and signaling hunger^17^. Given our finding that the deletion of MRAP2 leads to a decrease in food intake following a fast, we hypothesized that MRAP2 is important for the starvation-mediated activation of orexigenic AGRP neurons. To test this hypothesis we measured the expression of several markers of AGRP neuron activation in the ARC of ad libitum-fed or 24 h-fasted WT and *Mrap2* KO mice. We first measured cFos expression. For this, both satiated and fasted animals were sacrificed shortly after the start of the light phase and brains were harvested and sliced for immunofluorescence imaging. Brain slices were then stained for the expression of cFos, a transcription factor that is encoded by an immediate early gene and marks activated neurons. Neuronal activation in the ARC was quantified by counting cFos-positive cells for each condition and genotype. As previously demonstrated^18^, in WT animals the number of cFos positive neurons in this brain region is dramatically increased by fasting (Fig. 2a, b, e). In the *Mrap2* KO mice, however, the activation of AGRP neurons by fasting was almost completely prevented; no significant difference in cFos expression was detected between the ARC of fasted vs. fed *Mrap2* KO mice (Fig. 2c, d, e). A second readout of the activation of AGRP neurons is the expression of genes encoding the orexigenic neuropeptides AGRP and NPY; the expression of both neuropeptides in AGRP neurons is upregulated by fasting to promote food intake by either inhibiting MC4R or activating neuropeptide (NPY) receptors^19^. Whereas fasting resulted in a significant increase in *Agrp* and *Npy* gene expression in WT animals, this was not the case in *Mrap2* KO animals (Fig. 2f–i). Interestingly, the deletion of MRAP2 does not only affect AGRP neurons but also proopiomelanocortin (POMC) neurons since the decrease in *Pomc* expression caused by fasting is absent in *Mrap2* KO mice (Fig. 2j). Altogether, these results support the hypothesis that MRAP2 regulates central hunger sensing, since the deletion of MRAP2 impairs the starvation response in the ARC.

### MRAP2 is expressed in AGRP neurons

Although it is clear that MRAP2 is expressed in the hypothalamus, its expression within the AGRP neurons has only been shown at the transcriptional level^20^. For this reason we performed immunofluorescence detection of MRAP2, using a previously extensively validated anti-MRAP2 antibody^12^, in brain slices from mice expressing tdTomado in AGRP neurons (AGRP^CRE/tdTomato^ mice). We show that MRAP2 is expressed throughout the hypothalamus, including AGRP neurons (Fig. 2k–m).

### MRAP2 deletion in AGRP neurons impairs starvation sensing

**Fig. 3:**
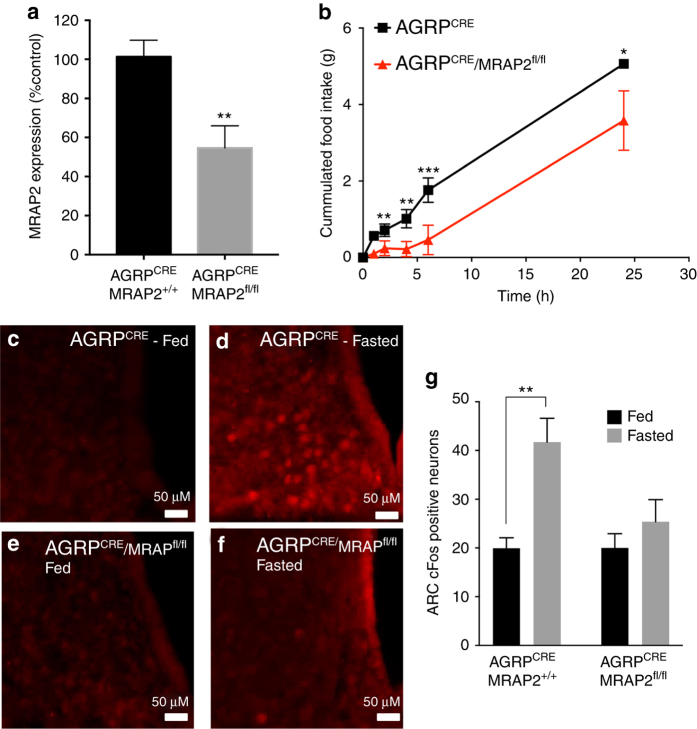
MRAP2 is important within AGRP neurons for starvation sensing. **a** qPCR measurement of MRAP2 expression in the ARC of AGRP^CRE^/MRAP2^+/+^ and AGRP^CRE^/MRAP2^fl/fl^ mice. **b** Food intake after a 24 h fast in AGRP^CRE^/MRAP2^+/+^ and AGRP^CRE^/MRAP2^fl/fl^ mice (*n* = 8 AGRP^CRE^/MRAP2^+/+^, *n* = 5 AGRP^CRE^/MRAP2^fl/fl^). **c**–**f** Immunofluorescent detection of cFos expression in the arcuate nucleus of fed or fasted AGRP^CRE^/MRAP2^+/+^ and AGRP^CRE^/MRAP2^fl/fl^ mice. **g** Quantification of fasting-induced cFos expression in the arcuate nucleus of AGRP^CRE^/MRAP2^+/+^ and AGRP^CRE^/MRAP2^fl/fl^ mice (*n* = 3/group). Error bars are SEM. ***p<0.001﻿, **p < 0.01, *p<0.05

To determine if the deletion of MRAP2 specifically in AGRP neurons is sufficient to prevent fasting-induced cFos expression in the ARC, we bred AGRP^CRE^ mice with MRAP2^floxed^ mice and compared cFos expression by immunofluorescence in the ARC of fed or 24 h-fasted AGRP^CRE^/MRAP2^+/+^ (control) or AGRP^CRE^/MRAP2^fl/fl^ mice. Consistent with a specific deletion of MRAP2 in AGRP neurons, the expression of MRAP2 in microdissected ARC from AGRP^CRE^/MRAP2^fl/fl^ mice is about half of that of AGRP^CRE^/MRAP2^+/+^ animals (Fig. 3a). Unlike the global *Mrap2* KO animals, AGRP^CRE^/MRAP2^fl/fl^ mice do not develop obesity (Supplementary Fig. 1). We found that like MRAP2 KO mice, AGRP^CRE^/MRAP2^fl/fl^ mice eat significantly less after a 24 h fast compared to the AGRP^CRE^/MRAP2^+/+^ control animals (Fig. 3b). Additionally, whereas fasting potently induced cFos expression in the ARC of AGRP^CRE^/MRAP2^+/+^ mice, no significant increase in cFos expression was measured in AGRP^CRE^/MRAP2^fl/fl^ animals (Fig. 3c–g). These results suggest that MRAP2 is required within AGRP neurons for their activation by fasting.

Although numerous circulating hormones inform the brain about the energy state, most suppress food intake (e.g. insulin, cholecystokinin, leptin, and peptide YY)^21^. By contrast, ghrelin is the only known circulating hormone that promotes food intake^22^. AGRP neurons express GHSR1a and are the main target of systemic ghrelin. Since fasting induces cFos activation in the ARC of GHSR1a KO mice (Supplementary Fig. 2), it is clear that a loss of ghrelin signaling does not result in a complete impairment of fasting-induced activation of AGRP neurons. However, due to the role of the ghrelin system in transmitting starvation signals to AGRP neurons and the regulatory function of MRAP2 on several GPCRs, we investigated how MRAP2 impacts GHSR1a function and signaling.

### MRAP2 interacts with GHSR1a in vitro

**Fig. 4:**
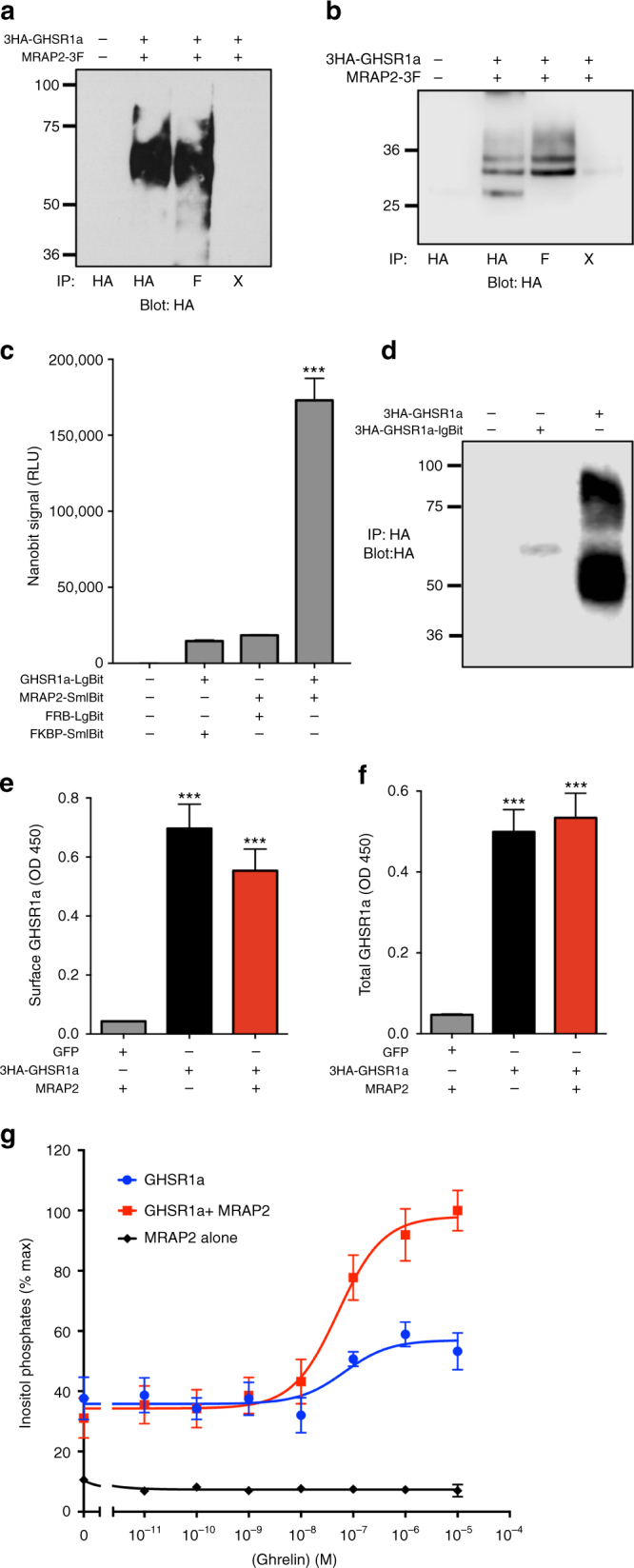
MRAP2 interacts with and potentiates the activity of the GHS-R1a. **a**, **b** Co-immunoprecipitation of lysates from HEK293T cells transfected with 3HA-GHSR1a and MRAP2-3Flag. **c** Measurement of GHSR1a and MRAP2 interaction in live cells using the NanoBit assay. **d** Western blot detection of 3HA-GHSR1a and 3HA-GHSR1a-LgBit transfection in CHO cells. **e** Surface expression of GHSR1a in CHO cells transfected with 3HA-GHSR1a with or without MRAP2. **f** Total expression of GHSR1a in CHO cells transfected with 2HA-GHSR1a with or without MRAP2. **g** Ghrelin-induced IP3 production as measured by IP1 accumulation in CHO-M1 cells transfected with MRAP2, 3HA-GHSR1a alone (empty vector), or GHSR1a with human MRAP2. Error bars are SEM. ***p < 0.001

We tested the ability of MRAP2 to form a complex with the ghrelin receptor using HEK293T cells transfected with 3 hemagluti﻿nin (HA)-tagged GHSR1a (3HA-GHSR1a) and 3xFlag-tagged MRAP2 (MRAP2-3F). Transfected cells were lysed and the GHSR1a or MRAP2 were pulled down using monoclonal anti-HA or anti-Flag antibody, respectively. Western blotting revealed reciprocal co-immunoprecipitation (Fig. 4a, b; full-size western blot shown in Supplementary Fig. 4). Specificity of the interaction was confirmed by the fact that in samples lacking the immunoprecipitating antibody (beads alone), neither GHSR1a nor MRAP2 was detected by western blotting. To further determine if MRAP2 and GHSR1a can form a complex in live cells while decreasing the risk of overexpression artefacts, we took advantage of the NanoBit protein–protein interaction assay. This assay relies on the recombination of two fragments, LgBit and SmlBit, of the bright NanoLuc luciferase fused to the proteins of interest. In this case we fused LgBit to the C-terminal region of 3HA-GHSR1a and SmlBit to the C terminus of MRAP2. The low intrinsic affinity of the NanoBit fragments for each other ensures that the dimerization of the proteins studied is required for the luciferase to reform. In addition, the brightness of the NanoLuc enzyme allows those protein–protein interaction experiments to be performed at low expression levels and thus prevent overexpression artefacts. This is achieved by driving the expression of the fusion proteins with a weak promoter (HSV-TK). We show that in mock transfected cells or in cells transfected with either GHSR1a-LgBit or MRAP2-SmlBit along with a non-interacting protein fused to the complementary fragment of NanoLuc (negative controls), very little luminescence is detected. In cells transfected with both GHSR1a-LgBit and MRAP2-SmlBit however, we are able to detect a very high luminescence signal (Fig. 4c), thus confirming that GHSR1a and MRAP2 can specifically form a complex in live cells. To verify that the expression level of 3HA-GHSR1a-LgBit used for the NanoBit assay was indeed lower than the GHSR1a driven by the cytomegalovirus (CMV) promoter used for the co-immunoprecipitation experiment, we transfected cells with either construct and assessed the expression level by western blot using anti-HA antibody (both constructs include an N-terminal 3XHA tag). While the CMV-driven 3HA-GHSR1a protein was readily detectable we were barely able to detect the HSV-TK-driven 3HA-GHSR1a-LgBit protein (Fig. 4d), thus confirming that GHSR1a and MRAP2 can specifically interact in live cells even at a low expression level.

Given that MRAP2 interacts with GHSR1a, we tested whether it could modulate the density of the receptor at the cell surface. To this end we transfected Chinese hamster ovary (CHO) cells with 3HA-GHSR1a and either MRAP2 or green fluorescent protein (GFP; as a control). We then fixed the cells and measured both cell-surface (in non-permeabilized cells) and total (in permeabilized cells) expression of 3HA-GHSR1a by fixed-cell enzyme-linked immunosorbent assay (ELISA). MRAP2 had no significant effect on either the cell surface density or total GHSR1a expression (Fig. 4e, f). Thus, MRAP2 does not appear to regulate trafficking of the ghrelin receptor.

### MRAP2 potentiates ghrelin-stimulated GHSR1a signaling

**Fig. 5:**
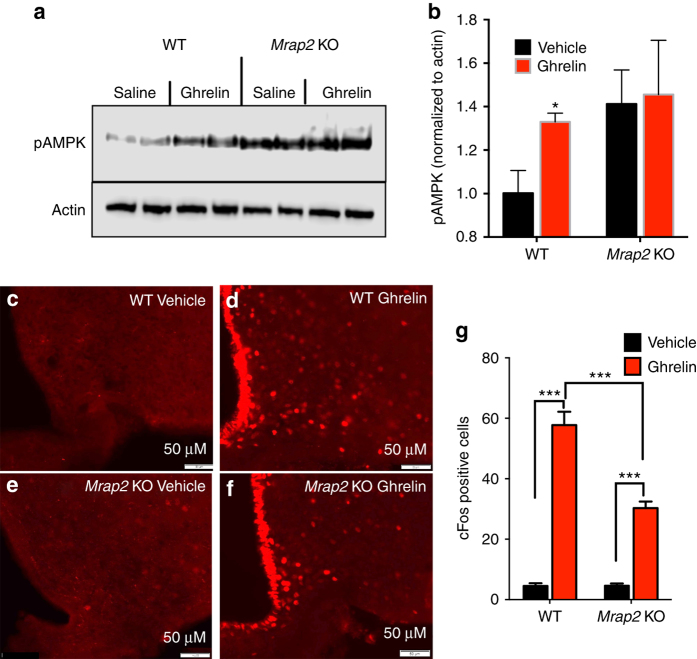
MRAP2 potentiates the activity of the GHS-R1a in the hypothalamus. **a**, **b** Western blot (**a**) and densitometry analysis (**b**) of AMPK phosphorylation and actin expression (loading control) in the ARC of WT and *Mrap2* KO mice injected IP with saline or ghrelin. *n* = 4/group. **c**–**f** Induction of cFos expression in ARC neurons of WT and *Mrap2* KO mice by ICV injection of saline or ghrelin. cFos expression in ARC neurons was detected by immunofluorescence in WT (**c**, **d**) or *Mrap2* KO (**e**, **f**) animals following ICV injection of saline or 13.5 μg ghrelin. cFos induction in each condition was quantified and represented as mean ± SEM (*n* = 5/group) (**g**). Error bars are SEM. *p < 0.05, ***p < 0.001

We next tested if MRAP2 modulates GHSR1a signaling. Since GHSR1a couples to the G-protein Gα_q/11_, we measured ghrelin-induced inositol triphosphate (IP3) production (reported by the concentration of accumulated inositol phosphates) in CHO cells expressing MRAP2, GHSR1a, or GHSR1a and MRAP2. As expected, ghrelin does not stimulate IP3 production in cells only expressing MRAP2. In cells expressing GHSR1a, we found that the efficacy of the ghrelin-induced IP3 response was greatly enhanced by the presence of MRAP2 (Fig. 4g). This finding demonstrates that MRAP2 potentiates GHSR1a activity, and suggests that the attenuated signaling downstream of GHSR1a in cells lacking MRAP2 may in part account for the defect in hunger sensing observed in MRAP2 KO mice. To test this hypothesis in vivo we measured the ability of ghrelin to activate AGRP neurons in the ARC of WT and *Mrap2* KO mice. To this end we injected ad libitum-fed WT and *Mrap2* KO animals with ghrelin or saline intraperitoneally before harvesting the ARC and measuring AMPK phosphorylation, a protein activated by ghrelin in AGRP neurons, by western blot. We found that as expected, ghrelin induced AMPK phosphorylation in WT animals (Fig. 5a, b). In *Mrap2* KO mice, however, the level of pAMPK was constitutively elevated but was not further increased by ghrelin, thus suggesting that ghrelin does not activate AGRP neurons in mice lacking MRAP2. To confirm this finding we performed intracerebroventricular (ICV) injections of ghrelin or saline in mice cannulated in the lateral ventricle of the brain and harvested the brains for immunofluorescence staining of cFos. Injection with ghrelin caused a significant increase in the number of cFos-positive neurons in the ARC compared to saline regardless of genotype. However, the response to ghrelin in *Mrap2* KO mice was about half of that in WT littermates (Fig. 5c–g), thus confirming that the deletion of MRAP2 causes a significant impairment of GHSR1a signaling in vivo and prevents the efficient activation of AGRP neurons by ghrelin.

### MRAP2 potentiates GHSR1a signaling in AGRP neurons

**Fig. 6:**
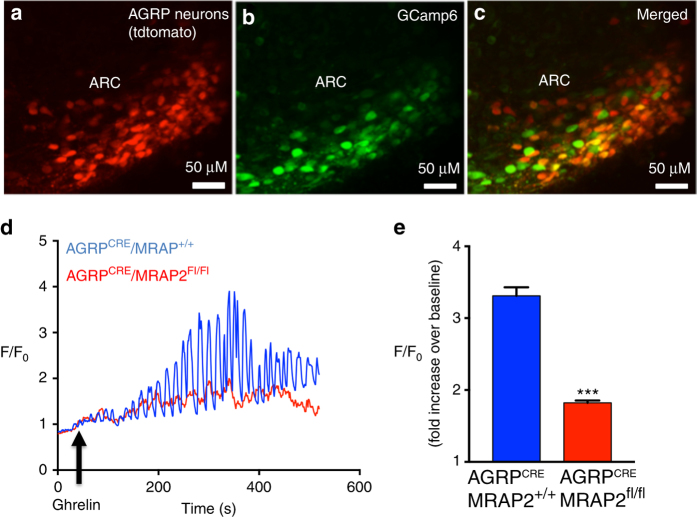
MRAP2 potentiates the ghrelin-stimulated calcium response in AGRP neurons. (**a**–**c**) Two-photon images of the ARC of a AGRP^CRE^ mouse injected with viruses coding for Cre-dependent tdTomato and GCamp6. **d** Representative traces of GCamp6 fluorescence intensity over time in AGRP neurons from AGRP^CRE^/MRAP2^+/+^ (*blue*) and AGRP^CRE^/MRAP2^FL/FL^ (*red*) mice. **e** Quantification of ghrelin-stimulated calcium responses in AGRP neurons from AGRP^CRE^/MRAP2^+/+^ mice (*n* = 5 mice/268 neurons) and AGRP^CRE^/MRAP2^Fl/Fl^ mice (*n* = 7 mice/415 neurons). Error bars are SEM. ***p < 0.001

To test whether the potentiation of ghrelin-stimulated signaling by MRAP2 observed in vitro takes place in AGRP neurons, we recorded the calcium response elicited by ghrelin in AGRP neurons of AGRP^CRE^/MRAP2^+/+^ and AGRP^CRE^/MRAP2^fl/fl^ mice. To this end, AGRP^CRE^/MRAP2^+/+^ and AGRP^CRE^/MRAP2^fl/fl^ mice were stereotaxically injected with adeno-associated viruses coding for CRE-dependent tdTomato and the GCamp6 calcium reporter unilaterally in the ARC. About 2 weeks after viral injection, brain slices containing the ARC were placed in the perfusion chamber of a two-photon microscope for fluorescence imaging (Fig. 6a–c). Slices were perfused with artificial cerebrospinal fluid (aCSF) followed by aCSF supplemented with 10 nM ghrelin, and GCamp6 fluorescence intensity was recorded over time in all AGRP neurons (tdTomato-positive) present in the field. At the end of the recording, slices were perfused with KCl to induce depolarization and allow the identification of all responsive neurons. We found that ghrelin induces calcium oscillations in AGRP neurons (Fig. 6d) with an average amplitude greater than threefold over baseline (Fig. 6e). Deletion of MRAP2 specifically in AGRP neurons significantly decreased the efficacy of ghrelin since the amplitude of the calcium response in AGRP neurons of AGRP^CRE^/MRAP2^fl/fl^ mice was below twofold over baseline (Fig. 6d, e). No difference in the overall number of AGRP neurons was measured in the ARC of AGRP^CRE^/MRAP2^+/+^ and AGRP^CRE^/MRAP2^fl/fl^ mice (Supplementary Fig. 3). This result demonstrates that, in agreement with our in vitro data, MRAP2 potentiates GHSR1a signaling in AGRP neurons.

### Ghrelin fails to stimulate food intake in MRAP2 KO mice

**Fig. 7:**
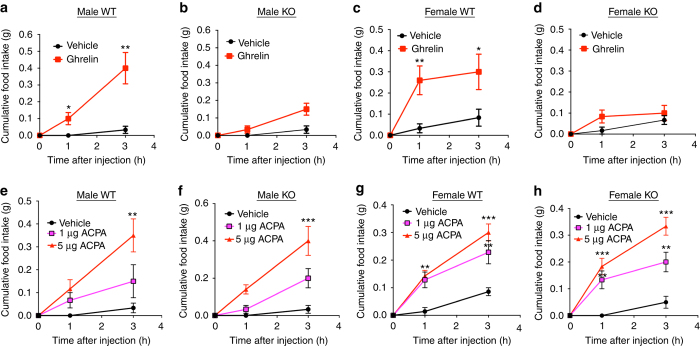
MRAP2 is necessary for ghrelin to induce food intake. **a**, **b** Food intake in satiated male WT (**a**) and *Mrap2* KO (**b**) mice after ICV injection of saline or 13.25 µg ghrelin (*n* = 6). **c**, **d** Food intake in satiated female WT (**c**) and *Mrap2* KO (**d**) mice after ICV injection of saline or 13.25 µg ghrelin (*n* = 6). **e**, **f** Food intake in satiated male WT (**e**) and *Mrap2* KO (**f**) mice after ICV injection of saline or 1 or 5 μg of the CB1R agonist ACPA (*n* = 5/group). **g**, **h** Food intake in satiated female WT (**g**) and *Mrap2* KO (**h**) mice after ICV injection of saline or 1 or 5 μg of the CB1R agonist ACPA (*n* = 5/group). Error bars are SEM. **p < 0.01, ***p < 0.001

It is well established that central injection of satiated mice with ghrelin causes a rapid and significant increase in food intake due to activation of hunger signaling. We reasoned that since MRAP2 is essential for GHSR1a signaling, *Mrap2* KO mice should have a diminished orexigenic response to ghrelin injection. To test this hypothesis, we cannulated the lateral ventricles of the brains of *Mrap2* KO mice and WT siblings. After recovery from the surgery, the animals were handled daily for a week in a manner that mimics ICV injections, to reduce the stress of the procedure. On the day of the experiment, satiated mice were injected with saline or ghrelin, and returned to their cages where food intake was monitored for 3 h. Whereas the injection of ghrelin in WT mice (both male and female) caused a significant increase in food intake compared to saline-injected animals (Fig. 7a, c), ghrelin injection in male and female *Mrap2* KO mice did not significantly stimulate food intake (Fig. 7b, d). This result confirms that the ghrelin receptor requires MRAP2 to trigger the behavioral response to hunger. To assess the specificity of the regulation of hunger through the GHSR1a by MRAP2, we tested the effect of MRAP2 on a distinct orexigenic pathway by activating the cannabinoid receptor 1 (CB1R)^23, 24^. To this end, satiated WT and *Mrap2* KO mice in which the lateral ventricle had been cannulated were injected with saline or with the CB1R agonist arachidonylcyclopropylamide (ACPA), and food intake was monitored for 3 h. ACPA caused a similar and significant increase in food intake compared to saline in both WT and *Mrap2* KO animals of both genders (Fig. 7e–h). This finding demonstrates that MRAP2 is not necessary for CB1R signaling in vivo, and that the lack of hunger sensing in *Mrap2* KO mice is due specifically to a defect in ghrelin signaling.

## Discussion

This study identifies a new role of MRAP2 in the regulation of energy homeostasis. We show that MRAP2 is important for fasting-mediated activation of AGRP neurons and that MRAP2 strongly potentiates ghrelin signaling. MRAP2 has previously been shown to potentiate MC4R signaling to promote leanness^10, 11^ and to inhibit the PKR1^12^, an anorexigenic GPCR expressed in POMC neurons^20^. This new role of MRAP2 in the ARC further establishes the importance of MRAP2 in the regulation of energy homeostasis by the hypothalamus. Although the deletion of MRAP2 significantly decreases fasting-induced AGRP neuron activation and food intake post food deprivation, those phenotypes are not found in GHSR1a KO mice (Supplementary Fig. 2)^25^, thus suggesting that they are not due to a loss of ghrelin signaling. It is however possible that developmental adaptations in the GHSR1a KO animal compensate for the absence of ghrelin signaling. Since we have shown that MRAP2 is not expressed in the embryo and only reaches normal expression levels around the time of weaning^11^, it is less likely that developmental adaptation take place in the *Mrap2* KO mice. However, because the homeostatic neuronal network can remodel and compensate for the ablation of AGRP neurons in neonates^8^, compensation in the *Mrap2* KO model cannot be ruled out.

We show that the deletion of MRAP2 does not cause a complete loss of ghrelin responsiveness both in vitro and in vivo since cells transfected with GHSR1a alone can elicit a small amount of IP3 production when stimulated with ghrelin, and direct ICV injection of ghrelin can induce some level of cFos expression in neurons of the *Mrap2* KO mice.

While this study focuses on the role of MRAP2 in AGRP neurons, we show that the fasting-induced decrease in POMC expression is also diminished in *Mrap2* KO mice. This may be due to the decreased inhibitory input from AGRP neurons onto POMC neurons or an indication that MRAP2 also plays an important role in POMC neurons where it is also expressed^20^.

*Mrap2* KO mice develop severe obesity over time^10, 12^, a phenotype that is in apparent contradiction with the conclusion of this study since one would predict that a decrease in GHSR1a signaling, due to the deletion of MRAP2, would promote leanness. It is however important to remember that, as stated earlier, MRAP2 is not a specific regulator of GHSR1a but also modulate the activity of MC4R^10, 11^, PKR1,^12^ and possibly other yet unidentified receptors involved in the regulation of energy homeostasis. Consequently, the phenotype of *Mrap2* KO mice is the result of a complex deregulation of the hypothalamic energy homeostasis machinery and does not reflect specifically the effect of *Mrap2* deletion on GHSR1a action. Later studies focusing on the manipulation of MRAP2 expression specifically in AGRP neurons will be necessary to determine the role of MRAP2 on the regulation of physiological actions of ghrelin.

This study also illustrates the promiscuity of MRAP2, which at first was thought to be a specific regulator of melanocortin receptors, and thus suggests that MRAP2 likely regulates several additional GPCRs. Identifying such GPCRs will improve our understanding of the global physiological roles of MRAP2. MRAP2 displays a high level of selectivity for the receptors it interacts with. For example, MRAP2 does not significantly modulate the activity of the β2-adrenergic receptor, the melanocortin-3 receptor, or NPY receptors^11^. In this study, the normal orexigenic response to ACPA suggests that MRAP2 is not required for CB1R function. The mechanisms involved in the selectivity of MRAP2 for a subset of GPCRs and its modulatory effects still remain to be elucidated.

Mice with a global or neuron-specific deletion of GHSR1a are resistant to diet-induced obesity and display improved insulin sensitivity^26, 27^, suggesting that inhibiting ghrelin actions could promote weight loss and decrease insulin resistance. Additionally, the stimulation of the ghrelin pathway is a promising approach for the treatment of cachexia^28^ and diabetic gastroparesis^29, 30^. Understanding how MRAP2 enhances the activity of GHSR1a activity may uncover new strategies to modulate ghrelin action and inform drug discovery efforts targeting GHSR1a.

## Methods

### Animals

C57BL/6N *Mrap2* KO and *Mrap2* floxed mice were generated by the Sanger Mouse Genetics Project. AGRP^CRE/tdTomato^ animals were generated by breeding AGRP^CRE^ (Agrp^tm1(cre)Lowl^ from the Jackson Laboratory) with R26-LSL-tdTomato reporter mice from the Jackson Laboratory. GHSR1a KO mice were kindly provided by Dr. Jeffrey Zigman (University of Texas Southwestern, Dallas, TX, USA). All animals were maintained in the University of Iowa temperature controlled animal facility with 12 h light/dark cycles (6 a.m./6 p.m.). Animals were fed with standard rodent diet (NIH-31) and allowed free access to water. All experiments using mice were approved by the Animal Care and Use Committee at the University of Iowa.

### Intracerebroventricular cannulation

Seven-week-old animals were anesthetized with intraperitoneal injection of a combination of xylazine and ketamine at dosage 10:100 mg/kg before being placed on a stereotaxic apparatus (David Kopf Instruments, Tujunga, CA, USA). After standard disinfection on surgical site, an incision was made to expose the skull and a small hole was drilled. A 26-gauge stainless steel cannula (PlasticOne) was placed into the left lateral ventricle (anteroposterior − 0.3 mm; medial lateral + 1.0 mm; dorsal ventral − 2.1 mm) and fixed in place with dental cement. Mice were allowed to recover for 7 days post surgery before experiments. At the end of the study, the localization of the injection site was confirmed by injecting 2 μl blue ink into the cannula.

### cFos immunofluorescence

Animals were deeply anesthetized with saturated isoflurane in a closed chamber and perfused with ice cold 0.1 M phosphate buffer solution (PBS; pH 7.4), followed by ice-cold 4% paraformaldehyde (PFA) in PBS. Whole brains were dissected out and post fixed in fresh 4% PFA for12 h at 4 °C. Brains were then immersed in 30% sucrose in PBS at 4 °C until they sunk. Samples were embedded in Tissue-Tek OCT compound (Sakura Finetechical Co., Ltd., Tokyo) and sectioned at 35 µm thickness using a cryostat (Leica Biosystems, Buffalo Grove, IL, USA). Free-floating sections were washed in PBS two times and incubated with blocking buffer containing 5% normal goat serum in PBS with 0.5% Triton X-100 for 1 h followed by rabbit polyclonal anti-cFos antibody (Catalog (Cat.) No. sc 52, Santa Cruz Biotechnology, TX, USA) in blocking solution (1:1000) overnight at 4 °C. After washed with PBS containing 0.1% Tween-20 (PBST), the sections were incubated in 1:500 Alexa Fluor 594 goat anti-rabbit IgG (R37117, Thermo Fisher Scientific) for 1 h at room temperature. The sections were washed in PBST five times and mounted onto superFrost slides (Fisher Scientific, Pittsburgh, PA, USA), air-dried at room temperature in a dark room and coverslipped with Prolong diamond antifade mountant with 4,6-diamidino-2-phenylindole (Molecular Probes by Life Technologies, Carlsbad, CA, USA). For cell counting, three different sections of the ARC of hypothalamus in each mouse were examined using × 20 magnification. cFos-positive cells were manually counted from entire ARC from five mice per group using the Olympus IX3 microscope system and Olympus Cellsens Dimension software.

### Cell culture and transfections

CHO-K1, HEK293T, and CHO-M1 cells were cultured in Dulbecco's Modified Eagle's medium (DMEM) /F-12 supplemented with 5% v/v fetal bovine serum and 1% penicillin–streptomycin solution. Cultures were incubated at 37 °C in a humidified atmosphere containing 5% CO_2_. Cells were transfected using LipoD293 in vitro transfection reagent (Signagen, Rockville, MD, USA). The total amount of DNA transfected for each condition within an experiment was kept identical.

### Co-immunoprecipitation and western blot

HEK293T cells were transfected with human MRAP2-3Flag and 3HA-GHSR1a using LipoD293 according to the manufacturer’s protocol. At 24 h after transfection, the cells were lysed with ice-cold non-denaturing lysis buffer containing 0.1% *n*-dodecyl-β-maltoside in PBS with proteinase inhibitor and incubated at 4 °C for 10 min on an orbital shaker, followed by centrifugation at 15,000 × *g* for 10 min. The protein lysates were transferred into new centrifuge tubes and following antibodies, Purified mouse anti-HA.11 epitope tag antibody (Biolegend, USA) or M2 mouse anti-Flag (Sigma-Aldrich, St. Louis, MO, USA), were added into the cell lysates at 1/5000 dilution. The protein lysates were then incubated on a shaker for overnight at 4 °C. 20 µl of protein G dynabeads (Life Technologies) were added into each sample and incubated at 4 °C for 1 h. The beads were washed four times with wash buffer and sample buffer containing LDS loading buffer with 5% β-mercaptoethanol was added on the beads to elude the target protein and antibodies and boiled 5 min. The same amount of protein from each sample was separated by SDS-polyacrylamide gel electrophoresis on 10% gel and transferred to polyvinylidene fluoride membranes. The proteins were detected by western blot using mouse anti-HA for GHSR1a co-immunoprecipitation experiments and mouse anti-Flag for detecting MRAP2. After 1 h in blocking solution containing 5% milk in PBS with 0.1 % Tween-20, the membranes were incubated with the primary antibody diluted 1/5000 in blocking solution overnight at 4 °C. The membranes were washed with PBST for 5 min three times and then incubated with secondary goat anti-mouse horseradish peroxidase (HRP) antibody (Cat. No. 170-6516, Bio-Rad Laboratories Inc, USA) at 1/5000 dilution for 45 min at room temperature followed by four washes with PBST. The WesternSure ECL substrate was applied on the membrane to detect the HRP-labeled secondary antibodies on the membrane and imaged on an cDigit scanner (LI-COR, NE, USA).

### NanoBit protein–protein interaction assay

CHO cells were transfected with GFP, 3HA-GHSR1a-LgBit + FKBP-SmlBit, FRB-LgBit + MRAP2-SmlBit, or 3HA-GHSR1a-LgBit + MRAP2-SmlBit. FKBP and FRB do not interact with GHSR1a and MRAP2, respectively, and were used to measure nonspecific NanoLuc recombination (Promega). Transfected cells were plated in a white opaque 96-well plate and allowed to attach overnight. Live-cell NanoBit substrate was added to the cells following the manufacturer’s direction and luminescence was measured 5 min later using a Spectramax i3 plate reader (Molecular Devices).

### Western blot for MRAP2-V5 or pAMPK from ARC

Ad libitum-fed or 24 h-fasted mice were deeply anesthetized with isoflurane before removing the brain. ARC was isolated by microdissection and solubilized in lysis buffer (38.5 mM Tris-HCl, 1 mM EGTA, 1 mM EDTA, 1% Triton-X 100, 1 mM sodium orthovanadate, 50 mM sodium fluoride, 5 mM sodium pyrophosphate, and 250 mM sucrose) with protease inhibitor on ice. Protein concentration was determined by bicinchoninic acid assay and the same amount of protein was loaded on polyacrylamide gels for SDS separation. Western blot was performed using either anti-V5 (Cell Signaling Technology, Rabbit mAb #13202) or anti-pAMPK (Cell Signaling Technology Rabbit mAb #2535).

### Quantitative PCR

After 24 h fasting, 11-week-old animals were deeply euthanized with saturated isoflurane in a closed chamber. Fresh whole hypothalami were collected into an ice-chilled tube and immediately frozen in dry ice and kept at −80 °C. Well-fed animals were used as control groups. Total RNA was extracted from hypothalamus using RNeasy lipid tissue mini kit (Qiagen, Germany) according to the manufacturer’s instruction and treated with RQ1 RNase-free DNase enzyme (Promega, USA). A unit of 1 µg cDNA was synthesized using iScript reverse transcription kit for reverse transcription (RT)-qPCR according to the manufacturer’s instruction (Bio-Rad, USA). Gene expression was quantified using mouse-specific predesigned probe-based quantitative PCR (qPCR) assays (IDT, USA) in 20 µl of PCR mixture containing 1 × KAPA Probe Fast qPCR master mix (Kapa Biosystems, MA, USA), 50 ng of cDNA (Bio-Rad), 200 nM of each primer, and FAM/BHQ-1 hydrolysis probe. Quantitative real-time PCR was done in optical 96-well plates on an iCycler (Bio-Rad) using a fast cycling protocol; 95 °C for 3 min followed by 40 cycles of 3 s at 95 °C and 20 s at 60 °C. The beta actin mRNA level was quantified as an endogenous control. The amount of cDNA in unknown samples was determined from a standard curve method. The target cDNA was compared to the amount of beta actin.

### Primer sequences

Primer sequences were as follows. Mouse *Mrap2*: primer 1, AGTCTGACACAAAGCTGTTCA; primer 2, GATTGGATTCTGGGTTGGTCT; probe, /56-FAM/TGTCTTCGT/ZEN/CAGCAAAGTCAGCACA/3IABkFQ/.

Mouse *Pomc*: primer 1, CTGTTCATCTCCGTTGCCA; primer 2, CATAGATGTGTGGAGCTGGTG; probe, /56-FAM/AGCGAGAGG/ZEN/TCGAGTTTGCAAGC/3IABkFQ/.

Mouse *Agrp*: primer 1, CAACAGCAGAACACAACTCAG; primer 2, GCACAAGAGACCAGGACATC; probe, /56-FAM/AGATCAGCA/ZEN/AGCAAAGGCCATGC/3IABkFQ/.

Mouse *Npy*: primer 1, ACAAGTTTCATTTCCCATCACC; primer 2, ACATCAATCTCATCACCAGACAG; probe, /56-FAM/CCCAGAACA/ZEN/AGGCTTGAAGACCCT/3IABkFQ/.

Mouse *β-actin*: primer 1, AGGTCTTTACGGATGTCAACG; primer 2, ATTGGCAACGAGCGGTT; probe, /56-FAM/ATTCCATAC/ZEN/CCAAGAAGGAAGGCTGG/3IABkFQ/.

### Immunofluorescent staining for MRAP2

We bred AgRP^CRE^ mice (#012899, Jackson Laboratories) with R26-LSL-tdTomato reporter mice to study the expression of Mrap2 protein in the AgRP neuron in the ARC of the hypothalamus. AgRP^CRE/tdTomato^ male mice were perfused with 4% PFA in 0.1 M phosphate buffer, pH 7.4, whole brains were dissected out and post-fixed in fresh 4% PFA overnight at 4 °C. The brains were cryopreserved by incubating in 30% sucrose in 0.1 M phosphate buffer. Cryosections were cut at 40 µm thickness and incubated in 10 mM sodium citrate at 80 °C for 30 min. The sections were then blocked in 5% normal goat serum/0.4% Triton X-100 in PBS for 1 h and subsequently incubated with rabbit anti-MRAP2 antibody (Novus Biologicals) 1:1000 in blocking buffer overnight at 4 °C. The sections were washed with 0.1% Tween-20 in PBS three times and incubated with goat anti-rabbit Alexa Fluor 488 antibody 1:300 for 1 h in blocking buffer and washed four times with 0.1% Tween-20 in PBS before mounting on a glass slide. Slides were visualized with an Olympus IX83 inverted fluorescence microscope.

### Inositol phosphate assay

Total inositol 1 phosphate (IP1) level was measured 48 h after transfection in adherent cells plated in a 384-well white opaque plate at a density of 14,000 cells/well. CHO-M1 cells (CHO cells stably expressing the muscarinic receptor) were transfected with MRAP2 alone, GHSR1a and empty vector, or GHSR1a and MRAP2 at a 1:5 ratio and plated in a 384-well plate the next day. IP1 accumulation was measured using the IP-One Tb HTRF kit (Cisbio Bioassays) according to the manufacturer’s instructions. Signals at 665 and 620 nm were detected using Spectramax i3 (Molecular Devices) equipped with a Cisbio HTRF module. Results were normalized to the signal obtained in response to 10 µM Carbachol for each condition.

### Food intake and energy expenditure measurement

For measurements of cumulative food intake, mice were individually housed in standard cages and, after 7 days of acclimation, food consumption was measured daily at 10.00 a.m. For energy expenditure, food intake, and activity measurements, 7-week-old male mice were individually housed in the home cage for 1 week before housed in CLAMS center feeder cages for 5 days. Data such as food intake, oxygen consumption, heat, and locomotion were collected automatically over a 5-day period and then analyzed with the Oxymax/CLAMS software. Daily food intake and activity data over the experimental period were collapsed to generate average daily food consumption and activity. Heat production was analyzed using analysis of covariance (ANCOVA).

### Fixed-cell ELISA

The CHO-K1 cells were cultured in a 24-well plate and transfected transiently with 3HA-GHSR1a and MRAP2, 3HA-GHSR1a and empty vector, or empty vector only. The fixed-cell ELISA was performed at 24 h after the transfection as described previously (eLife paper). After washing with PBS, the cells were fixed with 4% PFA in PBS for 10 min and washed with PBS twice, blocked with either 5% milk in PBS for surface-expression measurement or 5% milk in RIPA lysis buffer containing 150 mM NaCl, 50 mM Tris, 1 mM EDTA, 1% Triton X-100, 0.1% SDS, and 0.5% sodium deoxycholate, pH 8.0, for total receptor expression measurement for 45 min. Cells were subsequently incubated with 1/5000 mouse anti-HA monoclonal antibody in the blocking buffer for 2 h at room temperature and washed for 5 min three times with PBS followed by incubation with 1/5000 anti-mouse-HRP antibody for 1 h at room temperature before the cells were washed with PBS for 5 min three times. A volume of 200 µl of ELISA substrate (3,3ʹ,5,5ʹ-Tetramethylbenzidine, Sigma-Aldrich) was added into each well and reaction was stopped with 10% sulfuric acid when blue color was visible. Absorbance of ELISA reaction was measured at 450 nm using a Spectramax I3 plate reader.

### Stereotaxic injection

Mice were anesthetized in accordance with University of Iowa Carver College of Medicine guidelines. Mice were placed into stereotaxic frame and injection of 5 µl AAV1-GCamp6s virus and 5 µl AAV1-flox-tdTomato (UPenn vector core) were injected unilaterally into the ARC of the hypothalamus (bregma −1.6, M/L + 0.2, and D/V −5.4) with a flow rate of 1 µl/5 min. At completion of injection, needle was kept in injection site for an additional 15 min. Mice recovered from surgery for 7 days and tissue was processed for analysis 7–14 days after injection.

### Multiphoton imaging

Coronal slices (400 µm) from AAV1-GCamp6s/AAV1-flox-tdTomato-injected mice were prepared, in accordance with University of Iowa Carver College of Medicine guidelines. Slices were cut using a Vibratome 1000 Plus (Vibratome, St. Louis, MO, USA) in ice-cold slicing buffer (127 mM NaCl, 26 mM NaHCO3, 1.2 mM KH2PO4, 1.9 mM KCl, 1.1 mM CaCl2, 2 mM MgSO4, and 10 mM d-glucose) bubbled with 95% O2 and 5% CO2. Slices were then transferred to a holding chamber containing oxygenated aCSF (127 mM NaCl, 26 mM NaHCO3, 1.2 mM KH2PO4, 1.9 mM KCl, 2.2 mM CaCl2, 1 mM MgSO4, and 10 mM d-glucose) for 30 min at 34 °C and then for another 30 min at 22 °C for recovery, and subsequently transferred to a submersion recording chamber continually perfused with 32 °C oxygenated aCSF (rate: 2 ml/min). Slices were then placed in the Olympus FVMPE-RS multiphoton scanning microscope and equilibrated for at least 15 min before each recording. Baseline GCamp6 signal was recorded for the first 5 min and then slice was perfused with 10 nM ghrelin for 15 min. Peak fluorescence during the 15 min ghrelin perfusion was normalized to baseline measurements for each neuron recorded. At the end of the experiment, slices were perfused with high-potassium solution (127 mM KCl, 26 mM NaHCO3, 1.2 mM KH2PO4, 1.9 mM KCl, 2.2 mM CaCl2, 1 mM MgSO4, and 10 mM d-glucose) to induce depolarization and ensure cell viability.

### Effect of ghrelin and ACPA on food intake

Ghrelin-induced spontaneous food intake was observed in ad libitum Mrap2 KO mice and WO littermates during the light phase from 10 a.m. to 1 p.m. WT and *Mrap2* KO mice were cannulated in the lateral ventricle and individually housed. After recovering from the surgery, mice were acclimated to handling for 7 days before the behavioral test was started. Animals received a slow single ICV injection of either 3 μl of sterile normal saline, 13.5 μg rat ghrelin (Tocris Bioscience, Cat. No. 1465), 1 μg ACPA, or 5 μg ACPA (Tocris Bioscience, Cat. No. 1318) over a 1 min period using a 25 μl Hamilton syringe coupled to an injection cannula by a polyethylene tubing. After injection the injector was kept in place for 3 min to ensure full diffusion from the injector tip. Food intake was monitored at 0, 1, and 3 h after injection.

### Statistical analysis

Statistical difference was analyzed using GraphPad Prism 6 program (Graph Pad software, San Diego, CA, USA). The data were shown as mean ± SEM. One-way analysis of variance (ANOVA) followed by post hoc Tukey test or unpaired *t*-test or regular two-way ANOVA was used to determine statistically significant differences between the groups tested time points and different samples. Energy expenditure data were analyzed by ANCOVA. *p* < 0.05 was taken as the level of significance in all statistical analyses.

### Data availability

All data included in this study are available upon request.

## Electronic supplementary material

Supplementary information Supplementary Figures
