# Supplementary material for: MRAP2 regulates ghrelin receptor signaling and hunger sensing
Source: Nat Commun. 2017 Sep 28;8:713. doi: 10.1038/s41467-017-00747-6 (PMC5620068; doi:10.1038/s41467-017-00747-6)
Supplement: Supplementary file 1 — Supplementary information Supplementary Figures [file 41467_2017_747_MOESM1_ESM.pdf]

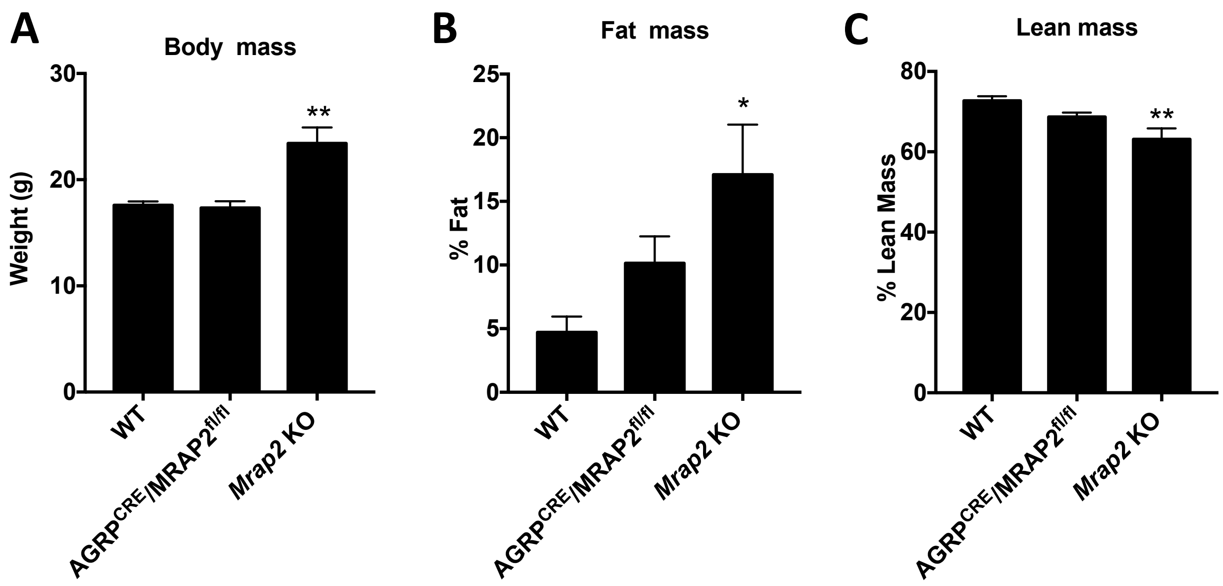

Supplementary Figure 1: AGRP^{CRE}/MRAP2$^{fl/fl}$ mice do not develop obesity. Body weight (**A**), and body composition (**B-C**) of 9-10 week old WT, AGRP^{CRE}/MRAP2$^{fl/fl}$ and MRAP2 KO female mice. n=4 per group. *$p<0.05$ ** $p<0.01$

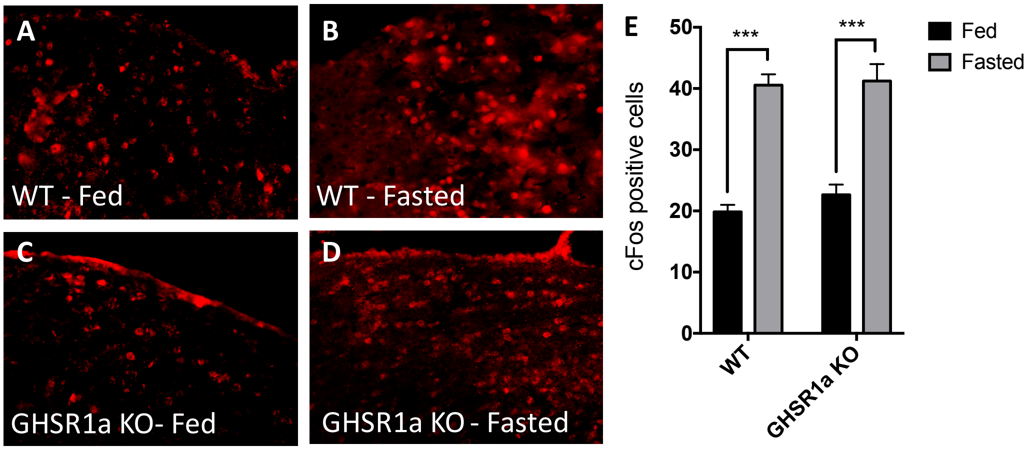

Supplementary Figure 2: GHSR1a is not required for fasting-induced cFos expression in neurons of the ARC. cFos expression in the arcuate nucleus of fed WT (**A**), 24h fasted WT (**B**), fed *GHSR1a* KO (**C**) and 24h fasted *Ghsr1a* KO (**D**) mice. **E.** Quantitative measurement of cFos activation in the arcuate nucleus of fed or fasted WT and *GHSR1a* KO mice. n=5 per group. *** p<0.001

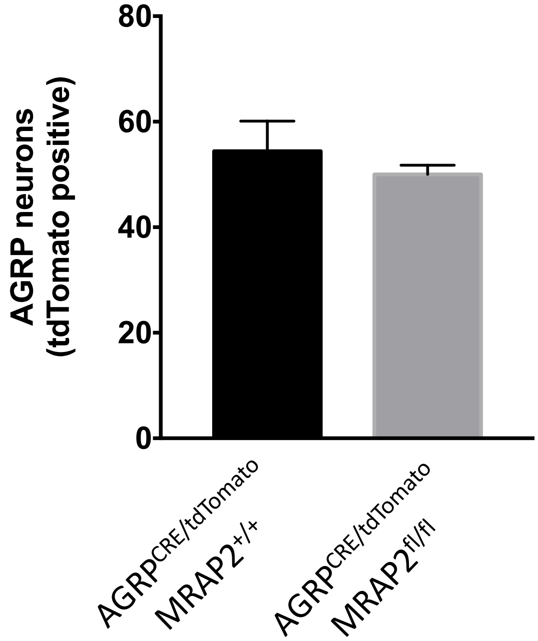

Supplementary Figure 3: Deletion of MRAP2 in AGRP neurons does not modify the number of AGRP neurons in the ARC. Histogram represents the average number of AGRP neurons in each brain slice containing the ARC from AGRP$^{CRE/tdTomato}$ / MRAP2$^{+/+}$and AGRP$^{CRE/tdTomato}$ / MRAP2$^{fl/fl}$. n=5 per group.

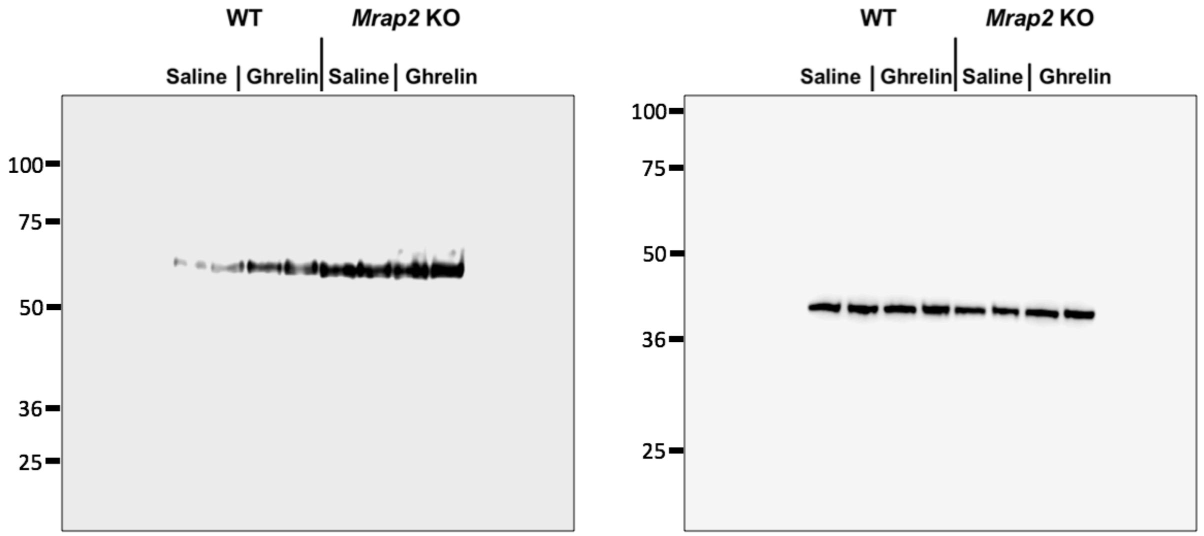

Supplementary Figure 4: Uncropped western blot for figure 5A
